# Effectiveness of novel fabrics to resist punctures and lacerations from white shark (*Carcharodon carcharias*): Implications to reduce injuries from shark bites

**DOI:** 10.1371/journal.pone.0224432

**Published:** 2019-11-18

**Authors:** Sasha K. Whitmarsh, Dhara B. Amin, John J. Costi, Joshua D. Dennis, Charlie Huveneers

**Affiliations:** College of Science and Engineering, Flinders University, Adelaide, South Australia; Institut de recherche pour le developpement, FRANCE

## Abstract

Increases in the number of shark bites, along with increased media attention on shark-human interactions has led to growing interest in preventing injuries from shark bites through the use of personal mitigation measures. The leading cause of fatality from shark bite victims is blood loss; thus reducing haemorrhaging may provide additional time for a shark bite victim to be attended to by emergency services. Despite previous shark-proof suits being bulky and cumbersome, new technological advances in fabric has allowed the development of lightweight alternatives that can be incorporated onto traditional wetsuits. The ability for these fabrics to withstand shark bites has not been scientifically tested. In this report, we compared two types of recently developed protective fabrics that incorporated ultra-high molecular weight polyethylene (UHMWPE) fibre onto neoprene (SharkStop and ActionTX) and compared them to standard neoprene alternatives. We tested nine different fabric variants using three different tests, laboratory-based puncture and laceration tests, along with field-based trials involving white sharks *Carcharodon carcharias*. Field-based trials consisted of measuring *C*. *carcharias* bite force and quantifying damages to the new fabrics following a bite from 3–4 m total length *C*. *carcharias*. We found that SharkStop and ActionTX fabric variants were more resistant to puncture, laceration, and bites from *C*. *carcharias*. More force was required to puncture the new fabrics compared to control fabrics (laboratory-based tests), and cuts made to the new fabrics were smaller and shallower than those on standard neoprene for both types of test, i.e. laboratory and field tests. Our results showed that UHMWPE fibre increased the resistance of neoprene to shark bites. Although the use of UHMWPE fibre (e.g. SharkStop and ActionTX) may therefore reduce blood loss resulting from a shark bite, research is needed to assess if the reduction in damages to the fabrics extends to human tissues and decreased injuries.

## Introduction

Although the risk of being bitten by a shark is intrinsically low [1], the occurrence of shark bites has increased globally in the last 30 years [1–4]. For example, the incidence of shark bites in Australia has increased from 1–3 per year in the 1980s to more than 10 per year in the 2010s [2]. Growth in human population, habitat modification and destruction, water quality, climate change and anomalous weather patterns, and the distribution and abundance of prey have all been proposed to explain this recent increase in the incidence of shark bites and shark bites per *capita* [2–5]. However, the infrequent occurrence of bites impedes our ability to assess the relative importance of causal factors that might have contributed to the rise in the number of bites (but see Afonso et al. 2017 and Meyer et al. 2018). It is likely that each location that is considered to be a ‘hotspot’ for shark bites has its own combination of factors contributing to the regional increase in shark bites [3, 4, 6].

Australia’s population has grown to over 25 million [7], with 85% of people living within 50 km of the coast [8]. Alongside this human population increase, there has also been an increase in water-based activities such as surfing, scuba-diving, snorkelling, swimming, and fishing, as well as easier access to remote locations [6]. Some of these activities are often considered to expose people to sharks, increasing shark bite risks [1]. For example, 16%, 33%, and 35% of shark bites in Australia between 1982 and 2011 occur when surfing, snorkelling, and scuba-diving respectively [1]. Other regions report higher incident rates for surfers, with 54% for South Africa [9] and 86% for Reunion Island [10]. Although previous studies have suggested that surfing may increase risk due to surfers resembling sharks’ natural prey [11], the prevalence of bites on surfers might also reflect their large amount of time spent in the water, be related to the time of day when most surfers are in the water (early morning and late evening), and the location of surf spots overlapping with shark space use.

While the chance of being bitten is low, fast-paced, news-driven media cycles generate wide-spread coverage of incidents and promote the use of a language of fear [12–14]. Subsequently, the public may think the likelihood of a bite is much greater than the actuality [13]. Due to these perceived and real safety concerns, a number of mitigation measures have been developed to reduce the risk from shark bites [15–17]. Shark bite mitigation measures typically consist of two broad approaches: area protection and personal protection. Area protection aims to reduce the probability of humans and sharks being in close proximity by reducing their spatio-temporal overlap, e.g. shark spotter programs [18], enclosures [19], and drone detections [20]. The limitations of area protection strategies (e.g. weather conditions affecting ability to see sharks, inapplicability in all regions) have given rise to the development of a range of personal shark deterrent devices designed to deter sharks that come in close proximity to humans (< 5 m) [16, 21]. In situations when area protection or personal deterrents are not able to prevent a shark bite from occurring, the ability to reduce the severity of injuries can also help in mitigating negative human-shark interactions. A chainmail suit was developed in early 1980s to reduce wounds from shark bites. However, the weight and restrained mobility of these suits can make them impractical. This has led to limited use of the suit aside for shark feedings during wildlife tourism. The need for a more lightweight and flexible fabric that can reduce injuries from shark bites has resulted in the development of new fabrics incorporated in neoprene aiming to reduce laceration and puncture injuries from shark teeth.

With millions of people exposed to risks in their working environments or while undertaking recreational activities, protective fabrics have been developed for a range of applications [22]. Ultra-high molecular weight polyethylene fibre (UHMPE; e.g. Dyneema^®^, Spectra^®^) are high performance polymer fibres (24) that are UV resistant and lightweight with low density (specific gravity = 0.97) [23]. UHMPE fibres are high modulus and strength, abrasion-resistant fibres, described as 10 times stronger than steel and 50% stronger than Kevlar [24]. Studies have shown that these fibres have a high cut, abrasion, and puncture resistance and can provide protection against lacerations [25, 26], and impacts [23, 25, 27–29]. Because of the lightweight and high-resistance characteristics, UHMPE fibres have been used for various forms of protective clothing (e.g. body armour, gloves, chainsaw protection, and fencing suits) along with mountain climbing gear and impact helmets [22, 30], showing promising potential as a protective fabric to reduce injuries from shark bites.

Injuries sustained from a shark bite can range from minor grazes and bumps to loss of limb or death. Most shark bites only cause minor injuries [9, 10, 31] as bites usually occur on limbs, with bites to the torso and damage to internal organs rare [32]. Yet, the global rate of fatalities has been increasing from 5 fatalities in 1982 to 14 in 2011, with an average of 13.4% of shark bites resulting in fatalities [1], despite improvements in emergency response time and medical knowledge. The primary cause of death in fatal shark bites is blood loss caused from vascular lacerations [9, 10, 31]. While the new fabrics may not prevent or lessen crushing injuries, it may reduce haemorrhaging, providing additional time for a shark bite victim to be attended to by emergency services [9, 10].

The ability of a shark bite to cause punctures and lacerations is partly dependent on the force exerted during a bite sequence. A few studies have investigated the mechanism and force of shark bites over the last 20 years [33–36]. Throughout these studies, bite force has been measured in various ways, either theoretically through anatomical dissection and subsequent construction of 3D models [37, 38] or through measurements taken from transducers gained through voluntary or forced bites, e.g. through stimulation of muscles with electric current [33, 39]. Two studies have investigated the bite force of white sharks *Carcharodon carcharias* using 3D models constructed from a CT scan of a 2.5 m white shark [37, 38]. However, studies have shown that theoretical bite force maximums are often higher than bite force recorded from live animals [34, 39] and the bite force of white sharks has never been recorded *in situ* and published in peer-reviewed literature.

Overall, the aim of this study was to assess the ability of new fabrics incorporated into neoprene to reduce injuries from white shark bites. We selected to test the fabric on white sharks because it is the species responsible for the most unprovoked bites (41%) [1] and fatalities (34%) [6] in Australia. Specifically, we determined whether more force was required to puncture and lacerate the new fabrics compared to standard neoprene, using custom-developed tests in the controlled conditions of a laboratory. However, laboratory-based testing does not replicate the kinematics of a bite, and so we wanted to compare the force required to puncture fabric with the bite force of *C*. *carcharias*. We, therefore, recorded the bite force of *C*. *carcharias in situ* and compared it to bite force estimated from kinematic models. Finally, we tested the ability of the new fabrics to reduce punctures from white shark bites by enticing white sharks to bite the new fabrics and standard neoprene, and comparing damages.

## Methods

### Materials tested

We used nine different fabrics (Table 1). Three were standard neoprene of varying thicknesses and were used as a control. The other six fabrics incorporated ultra-high molecular weight polyethylene (UHMWPE) fibre onto neoprene that may help mitigate injuries from shark-human interactions and were tested and compared to the control neoprene. Not all fabrics could be used in all tests due to availability. Thickness of the various fabrics tested also differed to account for the added thickness from the UHMPE. Most fabric was used in our first laboratory test (puncture test) to assess if neoprene thickness affected efficacy of UHMWPE. Fabrics used in the second laboratory test (laceration test) and in the field test were selected based on availability, marketability, and performance in the puncture tests. The UHMWPE fibre was either glued as a layer on top or on either side of the neoprene (referred to by manufacturers as SharkStop), or bonded into multiple layers between neoprene layers (referred to as ActionTX).

**Table 1:** Description of fabrics used in this study and tests in which they were used. Differences in tests used across fabrics were due to fabric availability. Commercial name of fabric provided in parentheses.

**Type**	**Thickness (mm)**	**Components**	**Test used**
**Control**	2	Standard neoprene lined with cotton	Puncture, laceration
**Control**	3	Standard neoprene lined with cotton	Puncture, field bite
**Control**	5	Standard neoprene lined with cotton	Puncture
**Test**	3	Single-lined UHMWPE (SharkStop)	Puncture
**Test**	3	Double-lined UHMWPE (SharkStop)	Puncture
**Test**	5	Double-lined UHMWPE (SharkStop)	Puncture, laceration, field bite
**Test**	3	400 g/m^2^ standard UHMWPE (ActionTX)	Puncture, field bite
**Test**	5	400 g/m^2^ ribbed UHMWPE (ActionTX)	Puncture
**Test**	5	800 g/m^2^ standard UHMWPE (ActionTX)	Laceration

### Laboratory testing of wetsuit material

#### Puncture tests

**Fig 1:**
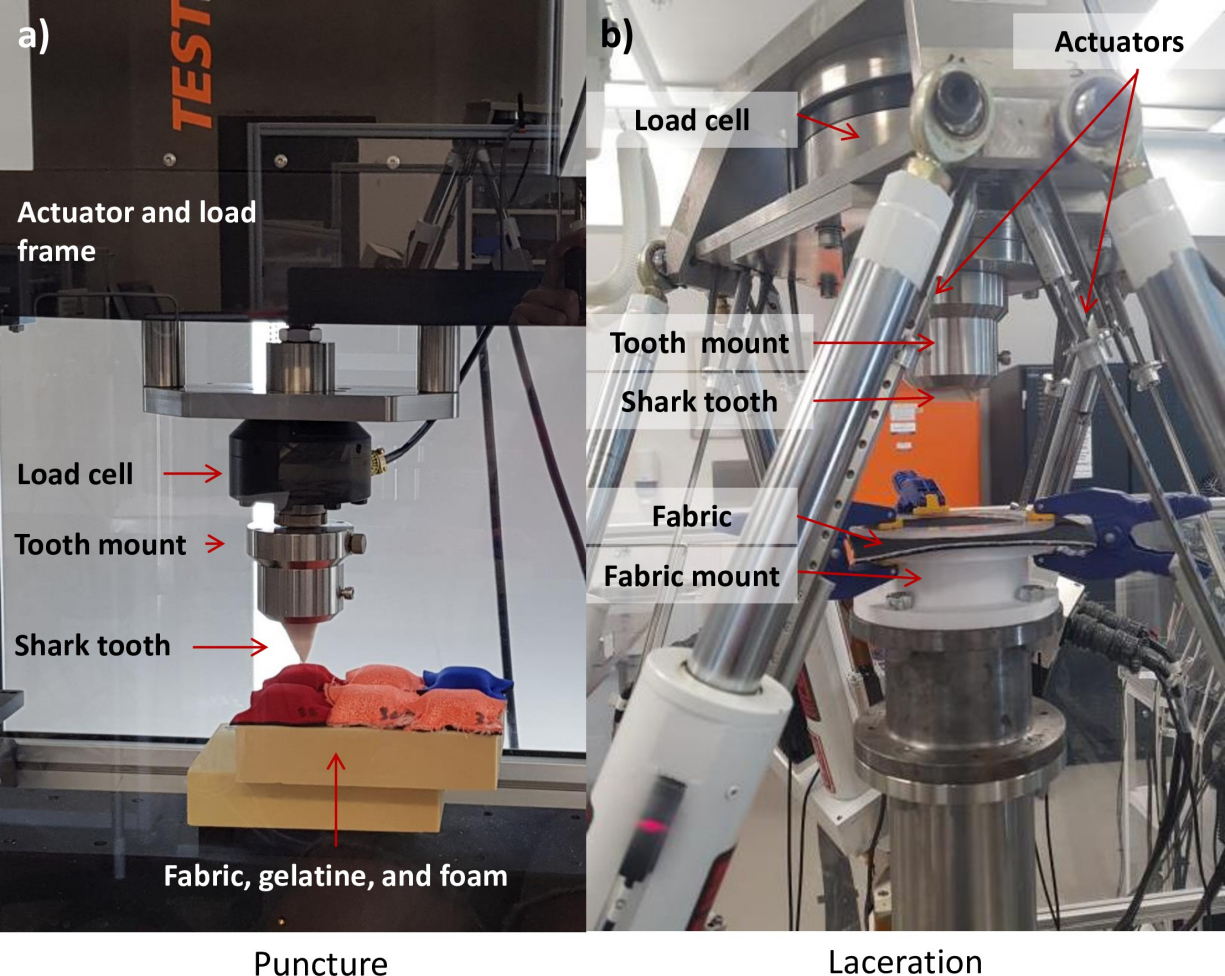
Set-up for a) the uniaxial Test Resources machine puncture tests and b) the six-axis hexapod robot laceration tests.

The force required to penetrate each fabric was tested using a TestResources 810LE Electrodynamic Test Machine (https://www.testresources.net/media/pdf/810LE.pdf) (Fig 1A). A 20% gelatine mix was made using distilled water and gelatine powder to replicate soft human tissue [40, 41]. The mix was heated to 38°C for two hours before being poured into 15 mm deep moulds and stored at 4°C for at least 12 hours. Squares (70 x 70 mm) of each fabric tested (Table 1) were stapled over a small square (30 x 30 mm) of the prepared gelatine mixture onto 20 density Sawbones foam (https://www.sawbones.com/products/biomechanical/biomechanical-test-materials/block-20-40-x-130-x-180mm.html). Sawbones foam was used to replicate bone under the skin and tissue [42]. Each piece of fabric was stapled on all four sides. This set-up allowed for the fabric to be secured while also mimicking a standardised and repeatable human flesh substitute as the gelatine has shown to be similar to human and pig skin tissue [40, 41] while the Sawbones foam is crafted to mimic human bone characteristics [42]. Gel depth of 15 mm was selected to enable the Sawbones foam to provide some resistance and ensure that puncturing of the fabric occurred. Fabrics were tested in a randomised order with each fabric having ten replicates. One shark tooth (36 mm wide × 37 mm high) mounted in acrylic (PMMA) was used for all testing in the Test Machine. Tests were run under the following settings: 25 mm penetration depth at a speed of 100 mm/s with the force required to reach this depth being recorded in Newtons. The action of this machine mimicked a straight downward stroke of a single bite. The maximum force was reached just before the teeth penetrated the fabric at which time the force rapidly decreased when the teeth penetrated the Sawbones foam. This maximum force was identified for each replicate and used to compare the force required to penetrate each fabric type. Control tests with Sawbones foam alone were conducted throughout the laboratory testing to assess if the tooth was becoming blunt. No evidence of decreases in penetration ability or bluntness of the tooth could be detected (S1 Fig).

#### Laceration tests

The force required to cut the fabrics in a sawing motion was tested using a six degree of freedom hexapod robot [43] (Fig 1B). The action of this machine was design to mimic the ‘head shake’ action white sharks often exhibit when biting prey [44]. A custom designed mount was used which affixed the fabric in a horizontal plane and consisted of a round 140 mm diameter plastic top with a matching polycarbonate ring. Each fabric piece (140 × 140 mm) was placed on the plastic top and secured with clamps and the polycarbonate ring. Emery paper was glued to the top of the mount and the underside of the ring which assisted in securing the fabric in place. Teeth were mounted so that the serrated edge was exposed horizontally. The sawing motion was now mimicked by the hexapod robot by applying ± 15 mm in the horizontal direction with a combined 40 N compressive force for 10 cycles at 0.5 Hz (sine waveform). The compressive force was applied to ensure the tooth cut into the fabric during the sawing translation. Three fabric types were tested based on the outcome of the puncture tests: two test fabrics (5 mm double-lined SharkStop and 5 mm 800 g/m^2^ ActionTX) and a control fabric (2 mm control neoprene). A minimum of ten replicate tests were conducted for each fabric type. Unlike the uniaxial testing, visible tooth bluntness was evident over time, thus, only six replicate tests were conducted using any one tooth. Analysis (described below) of replicates across test order and comparable cut sizes amongst replicates showed this number of replicates per tooth had no influence of tooth sharpness. We selected teeth of similar size ranging from 44 to 54 mm in width. The depth of cut, as a proportion of the total fabric thickness (due to the difference in fabric thickness across types), and the length of the cut produced were used to compare damage across the fabric types.

### Field-based testing

#### White shark bite force

The bite force of *C*. *carcharias* was measured at the Neptune Islands Group Marine Park using two Futek LTH350 2000 lb donut load sensors placed between two steel plates surrounded by foam. Sharks were encouraged to bite this set-up by attaching sections of southern bluefin tuna (*Thunnus maccoyii*) to the foam and allowing the tethered set-up to float behind the vessel. Before each sampling trip the force meter was calibrated with and without the steel plates and foam set-up using a hydraulic press. Maximum force was measured in N for each bite with the size of each shark visually estimated [45]. These bite values recorded from the field were adjusted using calculations from the laboratory calibrations with the following equation:

$$B_{true}=\frac{\left(B_{field}-c\right)}{m}$$

where *B*_*true*_ is the true bite force value in N, *B*_*field*_ is the bite force value recorded from the field, *m* is the slope of the line calculated from the calibrations (force recorded by pressure exerted) and *c* is the intercept. These field-based observations were then compared to theoretical maxima calculated from previous studies (Ferrara et al. 2011; Wroe et al. 2008; S1 Table).

#### Field testing of wetsuit fabric

The ability of the fabrics to reduce injuries from shark bites was also tested by enticing white sharks to bite the fabric covering dense foam wrapped around a 600 mm x 300 mm wooden board. New foam and fabric were used in each trial. The 3 mm control neoprene, 3 mm double-lined SharkStop, and 3 mm 400 g/m^2^ ActionTX were used for this testing, with 10–12 replicates performed for each fabric type. The order the fabric tested was block randomised. Bite sequences were filmed with the overall intensity, intensity of head shakes, and number of bites, recorded for each sequence. These factors were categorised on a scale from 1 to 3 as a subjective value from mild to severe by five independent experts with the average for each score used in analysis. The number, size, and depth of each cut on the fabric was counted or measured and compared, with depth calculated as a proportion of total fabric thickness. At least nine individual sharks ranging 3 to 4 m total length (TL) were involved in the field testing. Although shark identification could not be included in the analysis due to some bites occurring too fast for sharks to be identified, no single individual dominated the trials and the randomisation of fabric type ensured that results were not biased by individual sharks.

### Data analysis

Statistical analyses using PERMANOVA were performed using PRIMER v7 with the PERMANOVA+ add-on [46], while GLMM analyses were performed in the statistical program R 3.4.0 [47], with the packages *lme4* [48], *effects* [49], *car* [50], and *AICcmodagv* [51].

Univariate PERMANOVA analyses were conducted on resemblance matrices based on Euclidean distance to compare damages between the various fabrics. For the puncture tests, the force required to penetrate the fabric (N) was compared between fabric type (i.e. SharkStop, ActionTX, control; fixed with three levels) and individual fabrics (fixed with eight levels). For the laceration tests, cut length (mm) and depth (as a proportion of total thickness) were tested separately with the following individual factors: fabric type (fixed with three levels), position number (fixed with six levels), and tooth number (fixed with six levels). Position number refers to the order in which that fabric was tested within each tooth and was used to assess whether tooth bluntness affected the dependent variables. Tooth number refers to the tooth used for each test and was included in the models to account for differences across teeth. Number of cuts per replicate, average cut length per replicate, and average cut depth per replicate were compared between fabric types (fixed with three levels) for the field tests.

The difference in damages between fabrics were also tested using generalised linear mixed-effects models (GLMM) across the three response variables for the field tests: depth, length, and number of cuts. The error distribution and statistical family for each response variable was determined by visually examining their distribution and residuals. For each of the response variables, the models included combinations of fixed effect variables (fabric type) and random effect variables (intensity). Intensity was included as random effects to account for the different shark interactions between tests. Due to high correlations (>0.6; S2 Fig) between the three bite score metrics, only bite intensity was used in the models. Furthermore, we included replicates as a random effect in each model to account for non-independence within replicates. Relative support for each model was tested using the Akaike’s Information Criterion (AIC) for small sample sizes (AIC_c_), where the probability the model was ‘true’ increased with increasing AIC_c_ weight and decreasing ΔAIC_c_ [52].

## Results

### Bite force calculations

**Fig 2:**
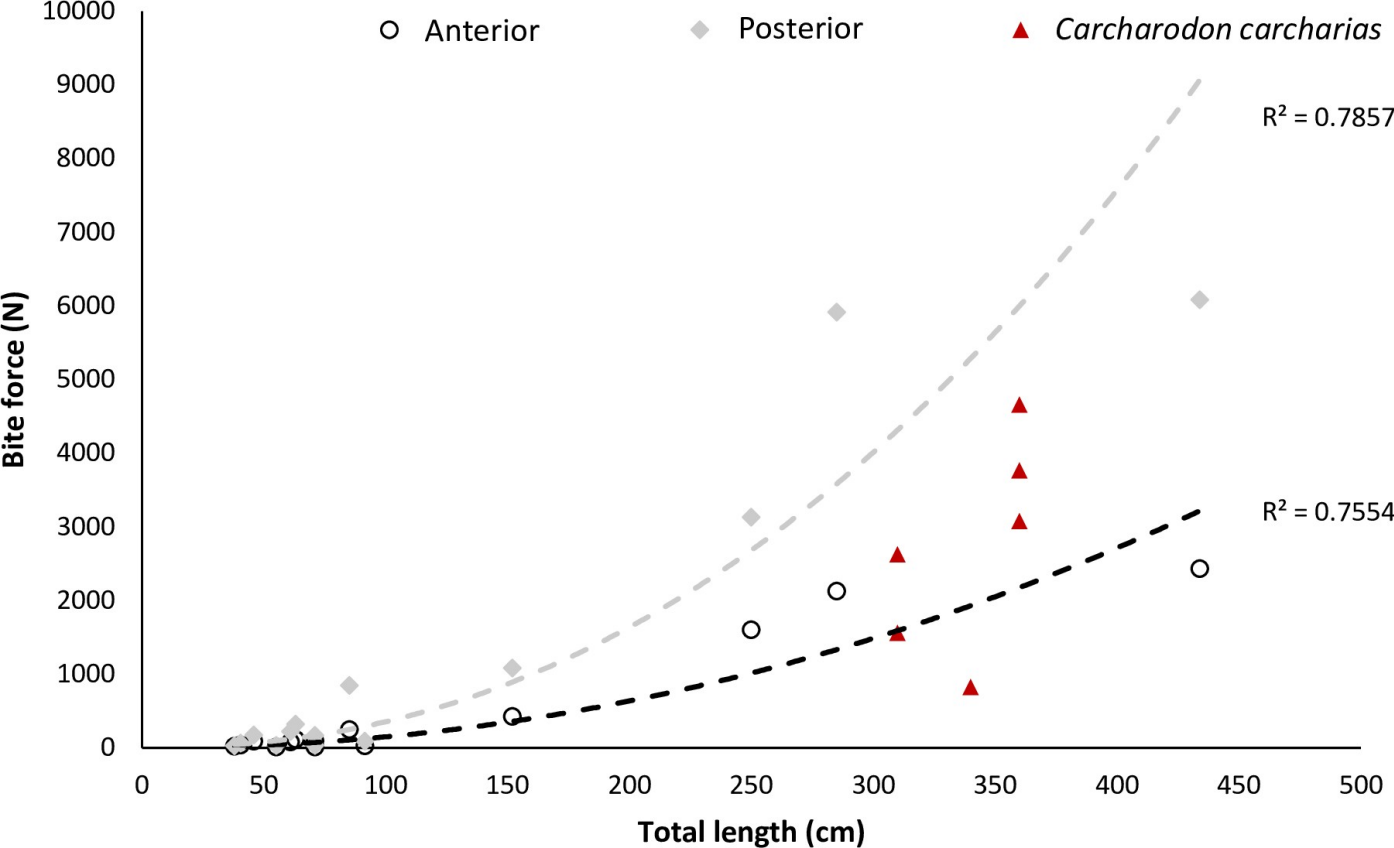
Relationship between total length of chondrichthyan and bite force for 13 species (listed in S1 Table). Source data: [34, 35, 38, 39, 53–58].

We recorded the force of six bites from *C*. *carcharias* ranging 3.1 to 3.6 m TL. Bite force recorded in the field ranged between 822–4657 N (mean ± standard error = 2751 ± 575). Previous studies calculated bite force using different methods with Wroe, Huber (38) and Ferrara, Clausen (37) using Finite Element Analysis to produce 3D models constructed from a CT scan of a 2.5 m TL *C*. *carcharias*, and from a modelled relationship between size and bite force calculated from a range of chondrichthyan species (posterior bite force = 0.0135 x size^2.2092^; anterior bite force = 0.0096 x size^2.0945^; Fig 2; S1 Table). Therefore, previous estimates of shark bite force are variable and range between 1011–1602 N for the anterior bite and 2678–3131 N for a posterior bite for a 2.5 m specimen across the three methods. Using the two thirds power rule, [38] also scaled the bite force values obtained from the 2.5 m specimen and calculated the theoretical maximum for a 6.4 m TL *C*. *carcharias* at 9320 N and 21367 N for the anterior and posterior bite respectively. Bite forces obtained using these models and those measured *in situ* were comparable (Fig 2).

### Puncture tests

**Fig 3:**
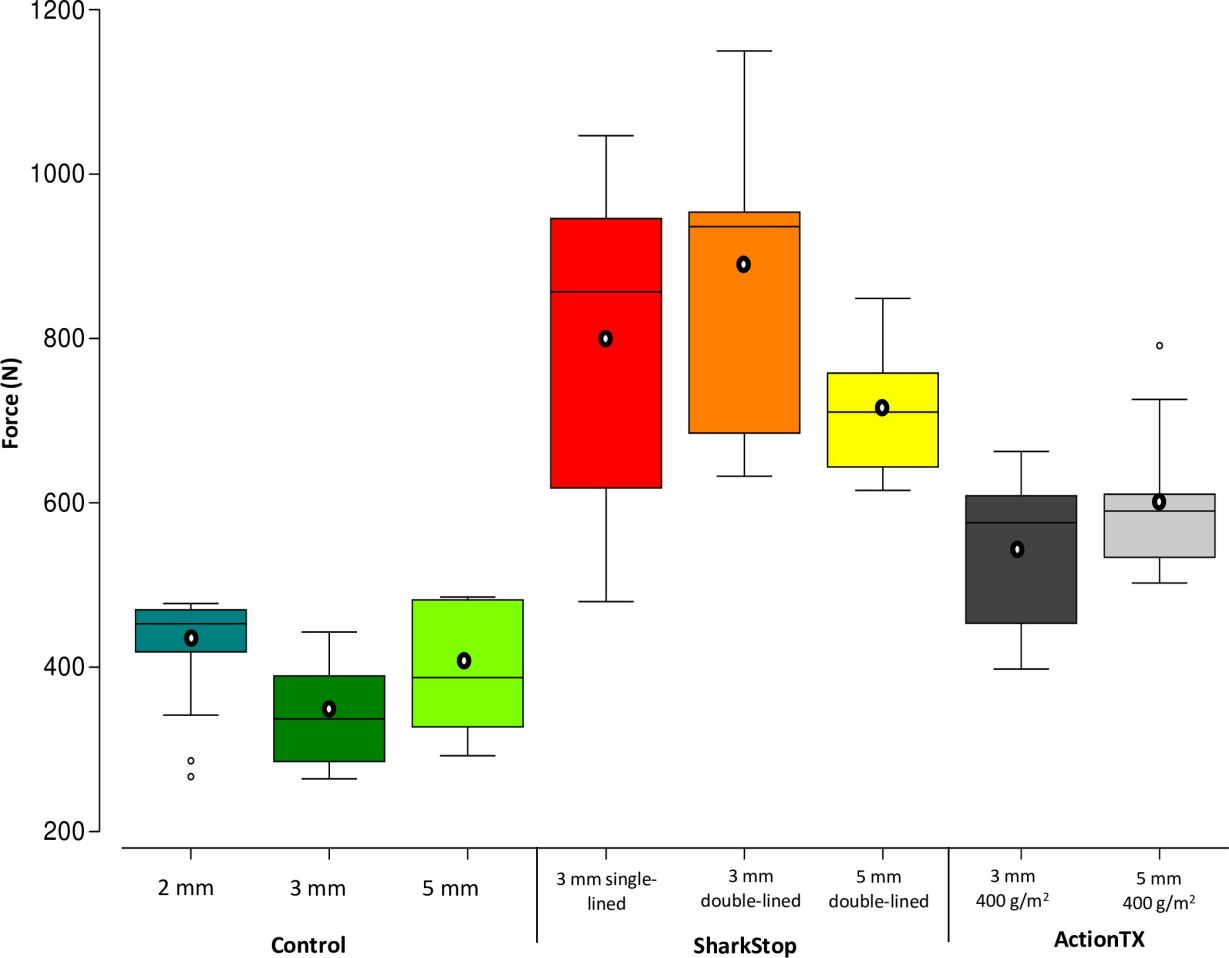
Box plots showing the minimum, interquartile range, median, and maximum of the force required to puncture each fabric (*n* = 10). White circles show the mean maximum force.

The force required to penetrate the different fabrics was variable and ranged from 264 N for 3 mm control neoprene) to 1150 N for the 3 mm double-lined SharkStop (Fig 3). Forces required to puncture each fabric were reasonably consistent across replicates (Fig 3), although the force necessary to puncture the 3 mm single- and double-lined SharkStop was more variable with a range of 567 and 517 N, respectively. The mean force required to puncture the fabric was similar between the three control fabrics tested (398 ± 75 vs. 338 ± 61 vs. 419 ± 77 N for 2, 3, and 5 mm respectively), but significantly different between fabric types (control vs. SharkStop vs. ActionTX; S2 Table), with SharkStop requiring the highest force to puncture the fabric and ActionTX requiring more force than control neoprene.

### Laceration tests

**Fig 4:**
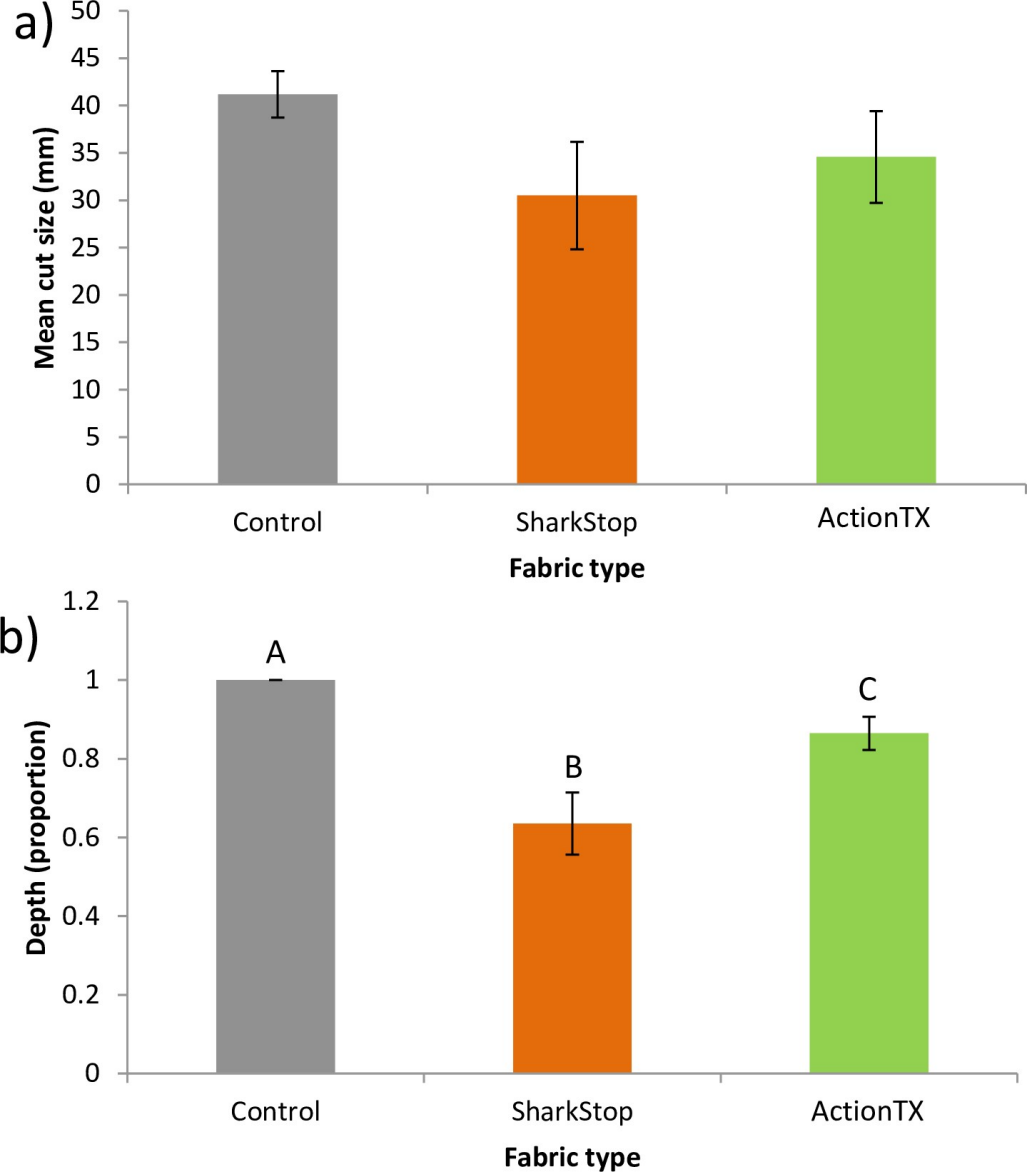
Changes in a) mean cut length (mm) and b) cut depth for each fabric type (*n* = 10). Letters indicate significant differences.

The mean length of the cut from the laceration tests was similar across fabric types (Fig 4; Table 2A), but the depth was significantly different across fabric types, with both test fabrics having shallower cuts than the control and SharkStop having the shallowest cuts (Fig 4; Table 2A). The order of testing (position) had no effect on either the cut length or depth (Table 2A), but the length of the cut was affected by teeth size (Table 2A).

**Table 2:** Univariate PERMANOVA analyses for a) the laceration tests for cut length (mm) and cut depth (proportion of total fabric depth) for the factors fabric type (SharkStop, Control, ActionTX), position number (order of test from 1–6), and tooth number (tooth used for testing from 1–6); and b) differences in the number of cuts, cut length, and cut depth between fabric types from the field-based tests. Unique permutations ranged from 12–999, where permutations were less than 100 denoted by *, Monte-Carlo values were used. Pairwise tests for position or tooth number were not performed. Bold values show significant values (< 0.05).

**a)**	**Laceration tests**					
Variable	Factor	Pseudo-*F*	*P*(perm)	Pairs	*t*	*P*(perm)
Cut length	Fabric type	1.23	0.266			
	Position #	0.85	0.513			
	Tooth #	6.55	**0.002**			
Cut depth	Fabric type	11.87	**0.001**	Control vs SharkStop Control vs ActionTX ActionTX vs SharkStop	4.40 3.08 4.40	0.002* 0.018* 0.002*
	Position #	0.48	0.81			
	Tooth #	1.35	0.24			
**b)**	**Field tests**				
Variable		Pseudo-*F*	*P*(perm)	Pairs	*t*	*P*(perm)
Number of cuts		0.25	0.783	-	-	-
Average cut depth		23.96	**0.001**	Control vs SharkStop	6.41	**0.001**
				Control vs ActionTX	6.28	**0.001**
				ActionTX vs SharkStop	0.10	0.920
Average cut length		3.07	**~0.05**	Control vs SharkStop	3.27	**0.003**
				Control vs ActionTX	1.17	0.272
				ActionTX vs SharkStop	0.98	0.377

### Field tests

Bite intensity was similar across fabric types (1.78 ± 0.1; Pseudo-F = 0.88, *P* = 0.44). However, the frequency distributions of bite intensities suggest that most bites on the standard neoprene (control) were <1.5 while most bites on the SharkStop were >1.5, justifying the inclusion of bite intensity in the GLMM.

**Fig 5:**
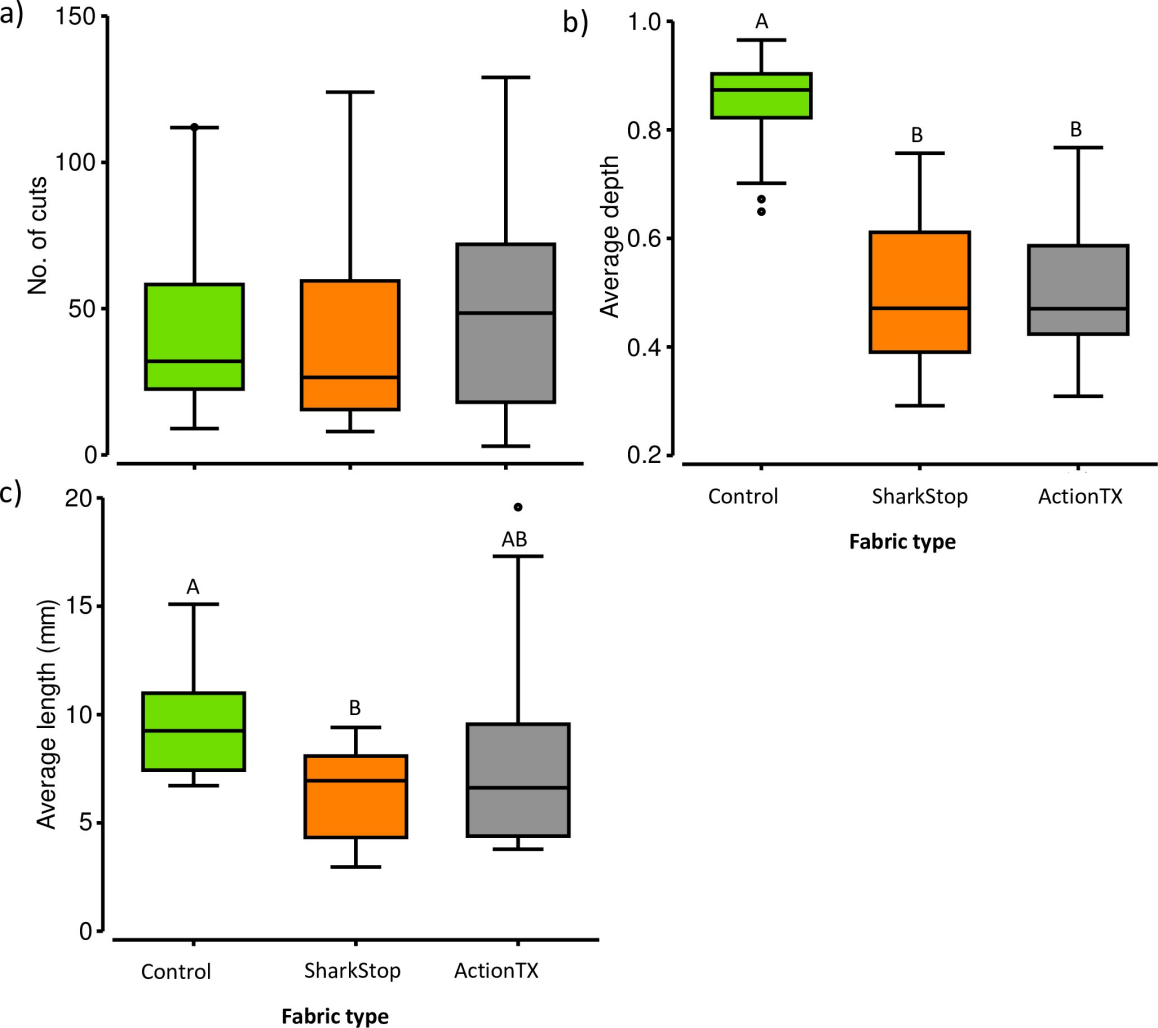
Boxplots showing the minimum, interquartile range, median, and maximum for a) the number of holes, b) hole depth as a proportion of total fabric depth, and c) the length of holes (mm) across fabric types. Letters indicate significant differences.

The univariate PERMANOVA (Table 2B) and GLMM (Tables 3 and 4) provided similar results, with fabric types affecting the size and depth of cuts, but not the number of cuts. The number of cuts per board ranged from 3 to 129 (mean = 46 ± 6) and did not vary between fabrics (Table 2B, Fig 5). The GLMM supported the univariate results and although the top-ranked model included fabric type and bite intensity (0.70 *w*AIC_c_; Table 3), SharkStop and ActionTX had low coefficient and neither were significantly different than the control neoprene (Table 4).

**Table 3:** Summary of models estimating the effects of fabric types (Types) on three variables (cut depth, cut length, and number of cuts) accounting for bite intensity (Intensity) as a random factor. Replicate was included in each model as a random factor. *k* = number of model parameters; AIC_*c*_ = Akaike’s information criterion corrected for small sample size; ΔAIC_*c*_ = difference in AIC_*c*_ between the current and the top-ranked model; *w*AIC_*c*_ = model probability. All models include replicates as a random effect.

Model	*k*	AIC_c_	ΔAIC_c_	*w*AIC_c_
**Depth ~ Types**	**4**	**1795.37**	**0**	**0.65**
Depth ~ Types + Intensity	5	1796.58	1.21	0.35
Depth ~ 1 (intercept-only)	2	1838.31	42.94	<0.01
**Length ~ Types + Intensity**	**6**	**9249.54**	**0**	**0.82**
Length ~ Types	5	9252.88	3.33	0.15
Length ~ 1 (intercept-only)	3	9256.21	6.67	0.03
**Cuts ~ Types + Intensity**	**6**	**-249.82**	**0**	**0.70**
Cuts ~ Types	5	-248.15	1.67	0.30
Cuts ~ 1 (intercept-only)	3	-229.73	20.09	<0.01

**Table 4:** Estimated coefficients (*β*) and their standard errors (SE) for each variable and factor, *z-*values of factors included in the top-ranked model (indicated for each variable), and the individual coefficient Type I error estimate (*P*). Bold values show significant values (< 0.05).

**Variable**	**Factor**	***β***	**SE**	***z*-value**	***P***
Depth	Intercept	2.06	0.28	7.24	**<0.001**
	ActionTX	-2.94	0.48	-6.12	**<0.001**
	SharkStop	-2.64	0.38	-6.86	**<0.001**
Length	Intercept	0.09	0.02	5.65	**<0.001**
	ActionTX	0.06	0.02	3.18	**0.001**
	SharkStop	0.08	0.02	3.86	**<0.001**
Number of cuts	Intercept	0.02	0.01	3.72	**<0.001**
	ActionTX	0.001	0.001	0.10	0.92
	SharkStop	0.005	0.001	0.67	0.50

The depth of cuts was smaller with the SharkStop and ActionTX (51 ± 10% and 55 ± 12% for SharkStop and ActionTX, respectively) than with control neoprene (85 ± 30%) (Table 2B; Fig 5). This was supported by the GLMM, with the top-ranked model including fabric type (0.65 *w*AIC_c_) and with SharkStop and ActionTX being significantly different to the control neoprene (Table 3). Bite intensity was not included in the top-ranked model (Table 3), suggesting that it did not affect the depth of cuts. The proportion of cuts which fully punctured the fabric showed similar trends with an average of 33 ± 5% of cuts fully punctured for ActionTX and 30 ± 5% for SharkStop compared to 78 ± 4% for control.

The length of cuts also varied across fabrics (Table 2B, Fig 5). Cuts in the control neoprene were larger (9.2 ± 2.7 mm) than in SharkStop (7.2 ± 2.1 mm). The average size of cuts in ActionTX (7.4 ± 2.4 mm) was in between those in control neoprene and SharkStop, and was not significantly different to either of these two other fabrics (Table 2B). However, the GLMM suggested some differences between ActionTX and control neoprene, but with a smaller coefficient (0.06) than that of SharkStop (0.08; Table 4). Bite intensity was also included in the top-ranked model (0.82 *w*AIC_c_; Table 3), indicating that the intensity of bites affected length of cuts.

## Discussion

This study showed consistent trends across the different tests and revealed that UHMWPE fibre incorporated onto neoprene is more likely to withstand damages from *C*. *carcharias* bites than standard neoprene. Laboratory tests undertaken in a controlled setting showed that SharkStop and ActionTX fabrics require a stronger force to be punctured and were less damaged by laceration trials than control neoprene. When tested in the field with 3–4 m TL *C*. *carcharias*, the neoprene with UHMWPE fibre were more resistant to bites than standard neoprene and beared less damage. There were little statistical differences between the two fabric types tested, but the SharkStop fabrics required a higher force to puncture than the ActionTX, which also had depth of cuts and punctures in-between those of the SharkStop and control neoprene. Our results showed that both fabrics tested may provide some protection against shark bites and could be used as part of a shark bite mitigation strategy if properly implemented.

Bite force measured *in situ* from 3.1–3.6 m TL *C*. *carcharias* revealed values up to 4657 N, which were mostly above the modelled maximum bite force from the anterior side of the jaws. This contrasts with previous studies in which voluntary bites of free-swimming sharks were lower than modelled maxima and electrically-stimulated bites from restrained live sharks [39]. Various factors may explain the discrepancy between recorded vs modelled bite force. Firstly, the bite forces we obtained may range between the anterior and posterior maxima due to the position of shark’s jaws on the force meter. As sharks were free-swimming the bites could be from any part of the jaw. However, based on videos of the bites, force sensors mostly seemed towards the anterior region of the shark’s jaws. Secondly, the relationship between bite force and size that was used to calculate the theoretical maxima was based on several chondrichthyan species and may not be accurate for white sharks. Thirdly, *C*. *carcharias* exhibit ambush hunting styles [59] that are unlike the bite strategies for other species and thus they may be more likely to bite near their maximum than other species.

The small number of *in situ* bites obtained on the bite force sensor was due to the difficult nature of enticing *C*. *carcharias* to bite a foreign object. It is also possible that the bite force sensor emitted some electric currents deterring *C*. *carcharias* as they got close to the bite force sensor. Indeed, we noticed several sharks abandoning their approach within less than 10 cm from the bite force sensor or as they touched it. The size range of *C*. *carcharias* biting the sensor was also limited (3.1–3.6 m TL), but these sizes are consistent with the size of *C*. *carcharias* responsible for most bites (>3 m) [6]. Continued testing with the bite force sensor and with sharks from a broader size range (e.g. < 3 m and > 4m TL) will provide additional estimates and further our knowledge of bite force values from a range of bite intensities and shark sizes.

The laboratory tests in a controlled environment showed promising results for the two types of fabric. The higher forces required to puncture SharkStop and ActionTX compared to standard neoprene may help prevent punctures from shark bites. Similarly these fabrics better withstand lacerating forces and reduce the depths of cuts compared to standard neoprene. For the 3 and 5 mm double-lined SharkStop, mean maximum force required to puncture the fabric (807 ± 208 and 874 ± 168 N) was similar to the lowest value recorded from the field (822 N) indicating it may be able to withstand weak bites without puncture. However, the controlled nature of the laboratory tests may not be directly comparable to actual shark bites. Damages incurred from a shark bites are not only due to a downward (as per the uniaxial machine) or side-to-side (as per the hexapod tests) force but relate to the bite kinematics [35, 37, 39, 59, 60]. As such, the force required to puncture the fabrics and the bite force measured *in situ* should be compared with caution.

In addition to the bite force which we measured, the size, strength, shape, and sharpness of the teeth [61–64], and the movements of the head and jaw [37, 39] are likely to influence the ability of a bite-proof wetsuit to reduce injuries from a shark bite. Bite intensity during the field testing was variable with some bites being considered as mild while others were classed as severe with sharks biting on the fabric several times, shaking its head, and dragging the equipment underwater. However, bite intensity was similar across fabric types and bite intensity was accounted for in the models by including it as a random effect in the GLMM. The severe nature of 27% of the bites was also useful to replicate extreme bites, showing that the SharkStop and ActionTX fabrics are able to perform better than standard neoprene during severe bites. Overall, across the field tests during which 3–4 m TL *C*. *carcharias* bit on the three fabrics, the number of cuts was the same across the three fabric types, but the cuts in the SharkStop and ActionTX fabrics were shallower and smaller (for the SharkStop) than in the standard neoprene.

While crushing injuries and skin tears without fabric puncture are still possible, our results suggest that these protective fabrics may help reduce blood loss providing more time for medical aid to be given to the victim. Blood loss is the leading cause of fatality from shark bites [9, 10, 31] and thus mitigation measures which minimise blood loss can contribute to reducing fatal shark bites.

Our results show that UHMWPE fibre may help mitigating injuries from shark bite and that it may therefore be beneficial to incorporate this fabric into wetsuit design. However, the weight and flexibility of the neoprene with this fabric (not tested as part of this study) might impede their applicability as they might decrease comfort or performance of the person wearing it. For example, surfers require light and flexible wetsuits to enable them to paddle efficiently. While the potential extra weight and lack of flexibility of these fabrics might hinder their use across a full wetsuit, it is possible to design wetsuit with these fabrics on specific panels, similarly to protective clothing aimed at reducing injuries from motorcycle accidents. The most suitable panels should be designed based on areas most often bitten, risk of haemorrhaging, and need for flexibility, which warrants further investigation [32]. While the present study showed that some fabrics can withstand shark bites better than others, future research should also investigate the magnitude of injury to flesh substitutes to determine to what extent these protective fabrics may reduce injuries. While we incorporated flesh mimics (e.g. foam and gelatine), the damages to these and the correlation between those and human injuries is unclear. Testing of the protective fabrics in an incorporated wetsuit design and the potential damage to flesh underneath would allow more robust recommendations and further promote the use of such shark bite mitigation measure. In conclusion, there were positive benefits for both fabric types against *C*. *carcharias* bites. These results were consistent across laboratory and field-based tests regardless of bite intensity. The inclusion of these fabrics into wetsuit designs may help reduce injuries from shark bites.

## Supporting information

S1 Table List of species and sources for which bite force was estimated (see Fig 1). (DOCX)

S2 Table Pairwise test from univariate PERMANOVA analysis. Main test results showed a significant difference between fabric (*Pseudo-F* = 28.71, *P*(perm) = 0.001) and fabric types (control, SharkStop, ActionTX; *Pseudo-F* = 85.46, *P*(perm) = 0.001). Unique permutations ranged 986–995.
(DOCX)

S1 Fig Force required to penetrate 5 mm into a 20 density Sawbones foam block to test if teeth bluntness occurred through the trials. The same tooth was used for all tests.
(DOCX)

S2 Fig Scatterplots showing the Pearson correlation coefficient for each combination of bite scoring metrics for all fabric types combined. (DOCX)
