# Supplementary material for: Effectiveness of novel fabrics to resist punctures and lacerations from white shark (Carcharodon carcharias): Implications to reduce injuries from shark bites
Source: PLoS One. 2019 Nov 18;14(11):e0224432. doi: 10.1371/journal.pone.0224432 (PMC6860444; doi:10.1371/journal.pone.0224432)
Supplement: S1 Table — (DOCX) [file pone.0224432.s001.docx]

**S1 Table.** List of species and sources for which bite force was estimated (see Figure 1).

| Common name | Scientific name | Source |
| --- | --- | --- |
| Bull shark | *Carcharhinus leucas* | D. Huber (pers. comm.) |
| White shark | *Carcharodon carcharias* | Wroe et al. (2008) |
| Blacktip shark | *Carcharhinus limbatus* | Huber et al. (2006) |
| Sharpnose sevengill shark | *Heptranchias perlo* | Huber (2006) |
| Horn shark | *Heterodontus francisci* | Huber et al. (2005) |
| Whitespotted bamboo shark | *Chiloscyllium plagiosum* | Huber et al. (2008) |
| Spotted chimaera | *Hydrolagus colliei* | Huber et al. (2008) |
| Giant chimaera | *Chimaera monstrosa* | J. Claes (pers. comm.) |
| Velvet belly lanternshark | *Etmopterus spinax* | J. Claes (pers. comm.) |
| Bonnethead shark | *Sphyrna tiburo* | Mara et al. (2010) |
| Blackmouth catshark | *Galeus melastomus* | J. Claes (pers. comm.) |
| Spiny dogfish | *Squalus acanthias* | Huber and Motta (2004) |
| Great hammerhead shark | *Sphyrna mokarran* | Huber et al. (2009)  Mara (2010) |
