# Supplementary material for: Effectiveness of novel fabrics to resist punctures and lacerations from white shark (Carcharodon carcharias): Implications to reduce injuries from shark bites
Source: PLoS One. 2019 Nov 18;14(11):e0224432. doi: 10.1371/journal.pone.0224432 (PMC6860444; doi:10.1371/journal.pone.0224432)
Supplement: S2 Table — Main test results showed a significant difference between fabric (Pseudo-F = 28.71, P(perm) = 0.001) and fabric types (control, SharkStop, ActionTX; Pseudo-F = 85.46, P(perm) = 0.001). Unique permutations ranged 986–995. (DOCX) [file pone.0224432.s002.docx]

**S2 Table.** Pairwise test from univariate PERMANOVA analysis. Main test results showed a significant difference between fabric (*Pseudo-F* = 28.71, *P*(perm) = 0.001) and fabric types (control, SharkStop, ActionTX; *Pseudo-F* = 85.46, *P*(perm) = 0.001). Unique permutations ranged 986–995.

| **Comparison** | **Pairs** | **t-value** | ***P*(perm)** |
| --- | --- | --- | --- |
| Within control | A, B | 2.58 | **0.018** |
|  | A, C | 0.63 | 0.528 |
|  | B, C | 1.94 | 0.072 |
| Within SharkStop | D, E | 0.80 | 0.458 |
|  | D, F | 1.29 | 0.223 |
|  | E, F | 2.69 | **0.021** |
| Within ActionTX | G, H | 1.26 | 0.196 |
| Control vs SharkStop | A, D | 5.51 | **<0.001** |
|  | A, E | 7.76 | **<0.001** |
|  | A, F | 8.39 | **<0.001** |
|  | B, D | 6.82 | **<0.001** |
|  | B, E | 9.45 | **<0.001** |
|  | B, F | 11.79 | **<0.001** |
|  | C, D | 5.85 | **<0.001** |
|  | C, E | 8.18 | **<0.001** |
|  | C, F | 9.16 | **<0.001** |
| Control vs ActionTX | A, G | 3.43 | **0.004** |
|  | A, H | 4.91 | **<0.001** |
|  | B, G | 6.05 | **<0.001** |
|  | B, H | 7.80 | **<0.001** |
|  | C, G | 4.06 | **0.003** |
|  | C, H | 5.59 | **<0.001** |
| SharkStop vs. ActionTX | D, G | 3.58 | **<0.001** |
|  | D, H | 2.91 | **0.012** |
|  | E, G | 5.36 | **<0.001** |
|  | E, H | 4.60 | **0.004** |
|  | F, G | 4.32 | **<0.001** |
|  | F, H | 3.12 | **0.004** |
