# Supplementary material for: Effectiveness of novel fabrics to resist punctures and lacerations from white shark (Carcharodon carcharias): Implications to reduce injuries from shark bites
Source: PLoS One. 2019 Nov 18;14(11):e0224432. doi: 10.1371/journal.pone.0224432 (PMC6860444; doi:10.1371/journal.pone.0224432)
Supplement: S1 Fig — The same tooth was used for all tests. (DOCX) [file pone.0224432.s003.docx]

*Supplementary material*

Effectiveness of novel fabrics to resist punctures and lacerations from white shark (*Carcharodon carcharias*): implications to reduce injuries from shark bites

Sasha K Whitmarsh, Dhara B Amin, John J Costi, Joshua D Dennis, Charlie Huveneers

Three replicate tests were run before, mid-way, and after the puncture tests and repeated for each of the four rounds of testing undertaken. No evidence of decreases in penetration ability or bluntness of the tooth could be detected within (before, mid-way, after) or between test round (1–4), or their interaction (univariate PERMANOVA: all *P* > 0.23; S1 Fig). Multiple rounds of testing had to be conducted due to fabric availability. No bluntness tests occurred mid-way for the fourth round due to the consistent results from the previous three rounds.


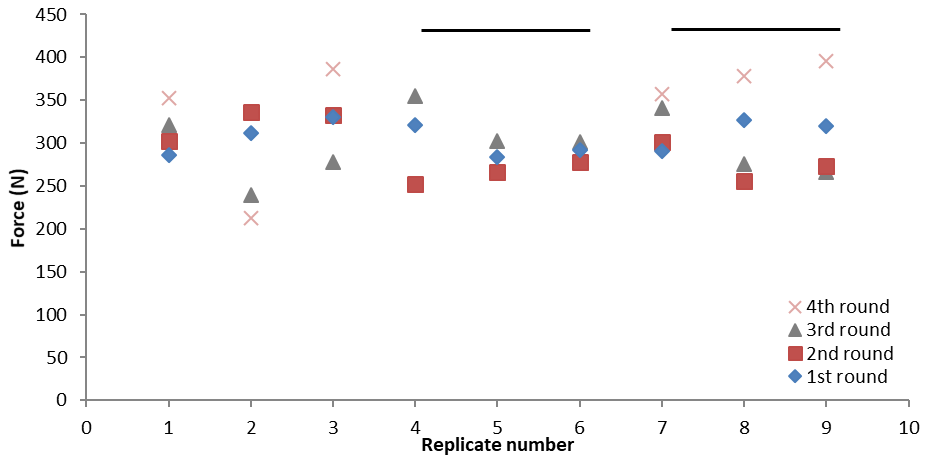


After

Mid-way

Before

**S1 Fig.** Force required to penetrate 5 mm into a 20 density Sawbones foam block to test if teeth bluntness occurred through the trials. The same tooth was used for all tests.
