# Supplementary material for: Effectiveness of novel fabrics to resist punctures and lacerations from white shark (Carcharodon carcharias): Implications to reduce injuries from shark bites
Source: PLoS One. 2019 Nov 18;14(11):e0224432. doi: 10.1371/journal.pone.0224432 (PMC6860444; doi:10.1371/journal.pone.0224432)
Supplement: S2 Fig — (DOCX) [file pone.0224432.s004.docx]

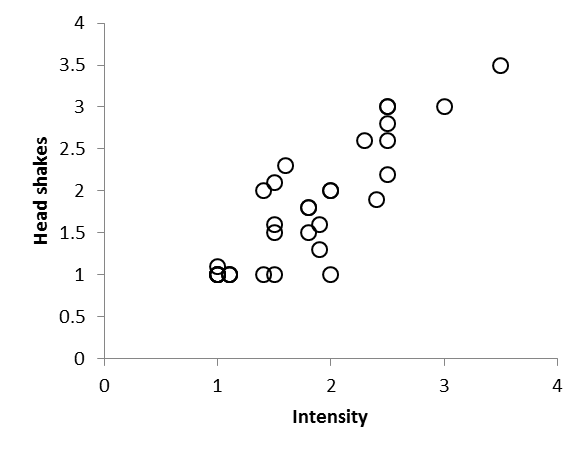

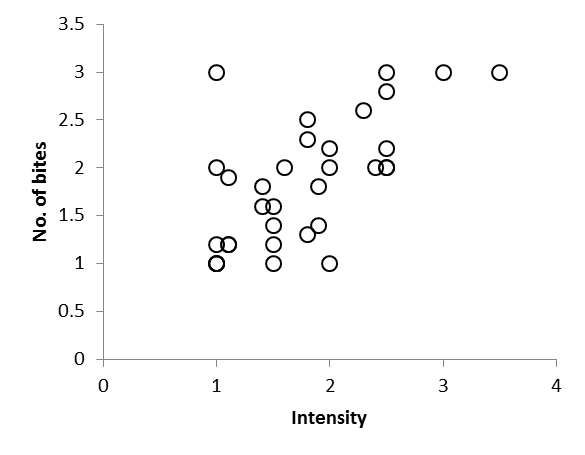

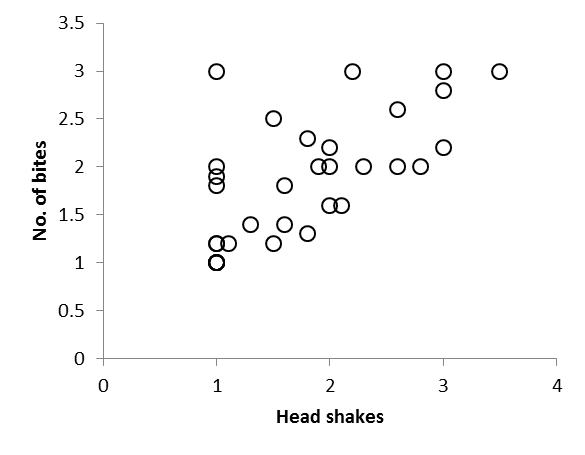


0.87

0.64

0.66

**S2 Fig.** Scatterplots showing the Pearson correlation coefficient for each combination of bite scoring metrics for all fabric types combined.
